# Metropolitan Hospital Sunday Fund, 1886

**Published:** 1886-12-18

**Authors:** 


### METROPOLITAN HOSPITAL SUNDAY FUND, 1886.

We extract the following interesting statement from the report of the above Fund, which was adopted at the meeting of the constituents held at the Mansion House on Monday the 13th inst., the Right Hon. the Lord Mayor, Sir Reginald Hanson, in the Chair.

"The fourteenth year of collecting this Fund has been marked by a new departure from the operations of former years, by the organisation of various public meetings in six divisions of the metropolis during the Hospitals Week immediately preceding Hospital Sunday, June 27th. At these meetings his Royal Highness the Duke of Cambridge, the Duke of Norfolk, the Duke of Westminster, the Marquess of Salisbury, the Earl of Dartmouth, the Earl of Northbrook, Sir Andrew Clark, Bart., M.D., Sir Sydney H. Waterlow, Bart., Mr. E. R. Cook, M.P., R. Brudenell Carter, Esq., F.R.C.S., President of the Medical Society of London, and many noblemen and gentlemen of eminence attended either as presidents or speakers. The special effort organised in the present year was the suggestion of the late James Wakley, Esq., M.D., Editor of the *Lancet*, accompanied by his generous offer to contribute a second donation to his like gift last year of £1,000, if four other gentlemen would each give an equal sum—a condition which was subsequently revoked.

"On 23rd March, the Council elected a special Committee, under the presidency of the Right Hon. Sir John Staples, K.C.M.G., Lord Mayor, to give effect to Dr. Wakley's suggestions; and Mr. Henry C. Burdett (acting for his friend Dr. Wakley, who was dangerously ill with cancer of the tongue) set himself earnestly to work with the Lord Mayor and Sir Edmund Hay Currie (hon. secretary), who all contributed largely, by securing most of the speakers, to crown this Committee's work with the success that has resulted from the meetings of 'The Hospitals Week.'

"The fund of 1866 amounts to £40,399 7*s*. 7*d*., as compared with £34,320 8*s*. 5*d*. in 1885, and an average of £30,121 6*s*. 8*d*. in the thirteen years preceding—the Rev. Canon Fleming, B.D., of St. Michael's, Chester Square, again heading the list of contributions in the sum of £1,006 4*s*.

"The working expenses have amounted to £1,592 15*s*. 3*d*., being 3.942 per cent. of the gross receipts, as compared with 3.474 per cent. last year, and with an average of 3.454 per cent. in the previous thirteen years; this comparatively small increase has arisen from the special effort in connection with the public meetings during 'The Hospitals Week.' The Council desire to express their thanks to the clergy and the ministers of all religious denominations for their valuable co-operation in pleading the cause of Hospital Sunday before their several congregations, with the marked success that has resulted this year."

It is gratifying to note the success which has attended the enterprise shown by the Council during the present year. As pointed out in another column, this new departure has been adopted in Leeds, and promises to be as great a success there as it has proved in the metropolis.

